# Characterization and in silico analysis of the domain unknown function DUF568-containing gene family in rice (*Oryza sativa* L.)

**DOI:** 10.1186/s12864-023-09654-1

**Published:** 2023-09-13

**Authors:** Kai Chen, Yilin Wang, Xiaoyan Nong, Yichi Zhang, Tang Tang, Yun Chen, Qikun Shen, Changjie Yan, Bing Lü

**Affiliations:** 1 College of Bioscience and Biotechnology, Yangzhou University, Yangzhou, 225009 Jiangsu China; 2 Key Laboratory of Plant Functional Genomics of the Ministry of Education, College of Agriculture, Yangzhou University, Yangzhou, 225009 China; 3 Jiangsu Province Engineering Research Center of Knowledge Management and Intelligent Service, College of Information Engineering, Yangzhou University, Yangzhou, 225127 Jiangsu China; 4 Jiangsu Key Laboratory for Eco-Agricultural Biotechnology Around Hongze Lake, Huaiyin Normal University, Huaian, 223300 China

**Keywords:** DUF568 domain, Expression pattern, Gene family, Phylogenetic analysis, Rice

## Abstract

**Background:**

Domains of unknown function (DUF) proteins are a number of uncharacterized and highly conserved protein families in eukaryotes. In plants, some DUFs have been predicted to play important roles in development and response to abiotic stress. Among them, DUF568-containing protein family is plant-specific and has not been described previously. A basic analysis and expression profiling was performed, and the co-expression and interaction networks were constructed to explore the functions of *DUF568* family in rice.

**Results:**

The phylogenetic tree showed that the 8, 9 and 11 *DUF568* family members from rice, *Arabidopsis* and maize were divided into three groups. The evolutionary relationship between *DUF568* members in rice and maize was close, while the genes in *Arabidopsis* were more distantly related. The cis-elements prediction showed that over 82% of the elements upstream of *OsDUF568* genes were responsive to light and phytohormones. Gene expression profile prediction and RT-qPCR experiments revealed that *OsDUF568* genes were highly expressed in leaves, stems and roots of rice seedling. The expression of some *OsDUF568* genes varied in response to plant hormones (abscisic acid, 6-benzylaminopurine) and abiotic stress (drought and chilling). Further analysis of the co-expression and protein–protein interaction networks using gene ontology showed that *OsDUF568 − *related genes were enriched in cellular transports, metabolism and processes.

**Conclusions:**

In summary, our findings suggest that the OsDUF568 family may be a vital gene family for the development of rice roots, leaves and stems. In addition, the OsDUF568 family may participate in abscisic acid and cytokinin signaling pathways, and may be related to abiotic stress resistance in these vegetative tissues of rice.

**Supplementary Information:**

The online version contains supplementary material available at 10.1186/s12864-023-09654-1.

## Background

Domains of unknown function (DUFs) are a group of protein families that are highly conserved yet uncharacterized. The first DUFs, DUF1 and DUF2, were identified and renamed the GGDEF domain and EAL domain by Chris Ponting in 1998 [1–3]. Since then, the number of known DUF families has increased rapidly owing to the sequencing of genomes in a large number of species. There are 19,632 families, of which, 4795 (24%) (out of 19 632) are DUF families according to the Pfam database version 35.0 [4]. Rice (*Oryza sativa* L.) is an important cereal crop, and DUFs are predicted to play important roles in its development and responses to abiotic stress [5–10].

DUF568 is a conserved domain that is exclusively found in plants. As of January 2023, the Pfam database contained a total of 1,713 sequences belonging to the DUF568-containing gene family (PF04526) across 150 species. An auxin-responsive protein AIR12 (Auxin induced in root cultures), which is a member of the DUF568 family, was reported to interact with other redox partners within the plasma membrane to establish a redox connection between the cytoplasm and the apoplast [11, 12]. In addition, *Os03g0194600*, also a member of the *OsDUF568* family, has previously been reported to be induced by nitrogen starvation in rice roots [13].

This study conducted a comprehensive genomic analysis of the *DUF568* gene family in rice, including phylogenetic analysis, subcellular localization prediction, cis-element and expression analysis. Then, the co-expressed genes and interacting proteins of the *OsDUF568* family were analyzed to reveal their potential biological functions. Furthermore, the expression of *OsDUF568* family in response to phytohormones and abiotic stresses were investigated through experiments. These results would provide valuable insights onto the *OsDUF568* family and pave the way for future research into its biological functions.

## Results

### Identification and phylogenetic analysis of *DUF568* family members

The DUF568 domain from Pfam was used to perform HMM (Hidden markov model) searches against the entire protein sequences of rice (*Oryza sativa*), maize (*Zea mays*) and *Arabidopsis thaliana* using local blast (E-value < 10^–15^). Eight, nine and 11 non-redundant genes were identified in rice, maize and *Arabidopsis*, respectively (Table 1 and Table S1). The eight DUF568-containing genes from rice were named as *OsDUF568.1* to *OsDUF568.8* according to their chromosomal order, which were found on chromosomes 3, 8 and 9. The length of OsDUF568 proteins ranged from 193 (OsDUF568.2) to 417 (OsDUF568.1) amino acids, and their predicted theoretical isoelectric points (pI) were concentrated between 8.52 (OsDUF568.8) and 9.74 (OsDUF568.3). The grand average of hydropathicity (GRAVY) scores were estimated to range from 0.08 (OsDUF568.2) to 0.44 (OsDUF568.7), indicating the hydrophilic nature of OsDUF568 proteins. The instability index (II) ranged from 31.05 (OsDUF568.1) to 45.21 (OsDUF568.4). OsDUF568.1, 3, 5 and 7 were predicted to be stable, while OsDUF568.2, 4, 6 and 8 were predicted to be unstable. The predicted subcellular localization showed that OsDUF568. 1, 2, 3 and 4 were located in the apoplast, while OsDUF568. 5, 6, 7 and 8 were targeted to the plasma membrane (Table 1). OsDUF568 proteins were annotated as “DUF568 (and Cytochrom-b561-FRRS1-like) domain-containing protein” according to RAP-DB (The Rice Annotation Project Database). Four members of OsDUF568 family, i.e., OsDUF568.1, 3, 5 and 7, were found to contain a Cytochrom-b561-FRRS1-like domain. In addition, OsDUF568.6 and OsDUF568.8 were recognized as AIR12 [11]. The results suggested that OsDUF568 proteins may have diverse structure and different functions.

**Table 1:** *OsDUF568* gene family and the predicted protein properties

Gene Name	Gene Symbol	MSU Locus	RAP Locus	Chr	Exons	Protein (aa)	MW (Da)	Theoretical pI	II	GRAVY	Predicted Location	Annotation
OsDUF568.1	LOC4331925	LOC_Os03g09850	Os03g0194300	3	1	417	43,733.55	9.54	31.05	0.34	apoplast	DUF568 and Cytochrom-b561-FRRS1-like domain-containing protein
OsDUF568.2	LOC4331928	LOC_Os03g09880	Os03g0194600	3	1	193	19,873.37	9.62	41.45	0.08	apoplast	DUF568 domain-containing protein
OsDUF568.3	LOC4331929	LOC_Os03g09900	Os03g0194900	3	2	384	41,057.01	9.74	35.45	0.15	apoplast	DUF568 and Cytochrom-b561-FRRS1-like domain-containing protein
OsDUF568.4	LOC4345306	LOC_Os08g24790	Os08g0335600	8	1	204	20,620.28	9.24	45.21	0.21	apoplast	DUF568 domain-containing protein
OsDUF568.5	LOC4346084	LOC_Os08g41280	Os08g0524200	8	3	390	40,893.24	9.63	33.03	0.27	plasma membrane	DUF568 and Cytochrom-b561-FRRS1-like domain-containing protein
OsDUF568.6	LOC4346085	LOC_Os08g41290	Os08g0524400	8	1	263	25,356.88	9.23	43.60	0.33	plasma membrane	DUF568 domain-containing protein; AIR12
OsDUF568.7	LOC4347483	LOC_Os09g32470	Os09g0500900	9	3	394	40,983.54	9.62	36.14	0.44	plasma membrane	DUF568 and Cytochrom-b561-FRRS1-like domain-containing protein
OsDUF568.8	LOC107276412	NONE	Os09g0501100	9	1	291	29,248.53	9.46	43.47	0.19	plasma membrane	DUF568 domain-containing protein; AIR12

*Chr* Chromosome, *MW* Molecular weight, *pI* Isoelectric point, *II* Instability index, *GRAVY* Grand average of hydropathicity

**Fig. 1:**
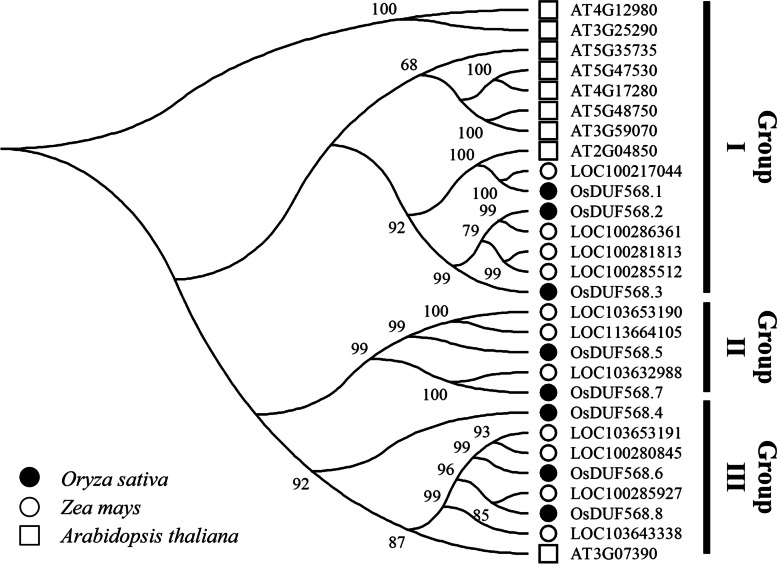
Phylogenetic tree showing the evolutionary relationships between DUF568 proteins from rice, Arabidopsis thaliana and maize. The major three phylogenetic clusters were marked as I, II and III based on genetic distance. There were eight, 11 and nine DUF568 proteins from rice (filled circles), maize (unfilled circles) and Arabidopsis thaliana (unfilled square), respectively

To extend our understanding of *OsDUF568* family, neighbor joining tree of *DUF568* homologous genes from rice, maize and *Arabidopsis thaliana* was constructed by bootstrap method (Fig. 1). These DUF568 members were classified into three groups (I, II, III). In three groups, the number of DUF568 members in rice and maize distributed evenly, while eight of nine DUF568 members from *Arabidopsis thaliana* gathered in Group I, and the remaining one gene (AT3G07390) belonged to Group III. The classification results showed that genetic distance of *DUF568* between rice and maize was close, genes in *Arabidopsis* were far away and more conservative.

### Analysis of protein sequences, conserved domains and motifs of OsDUF568 family

**Fig. 2:**
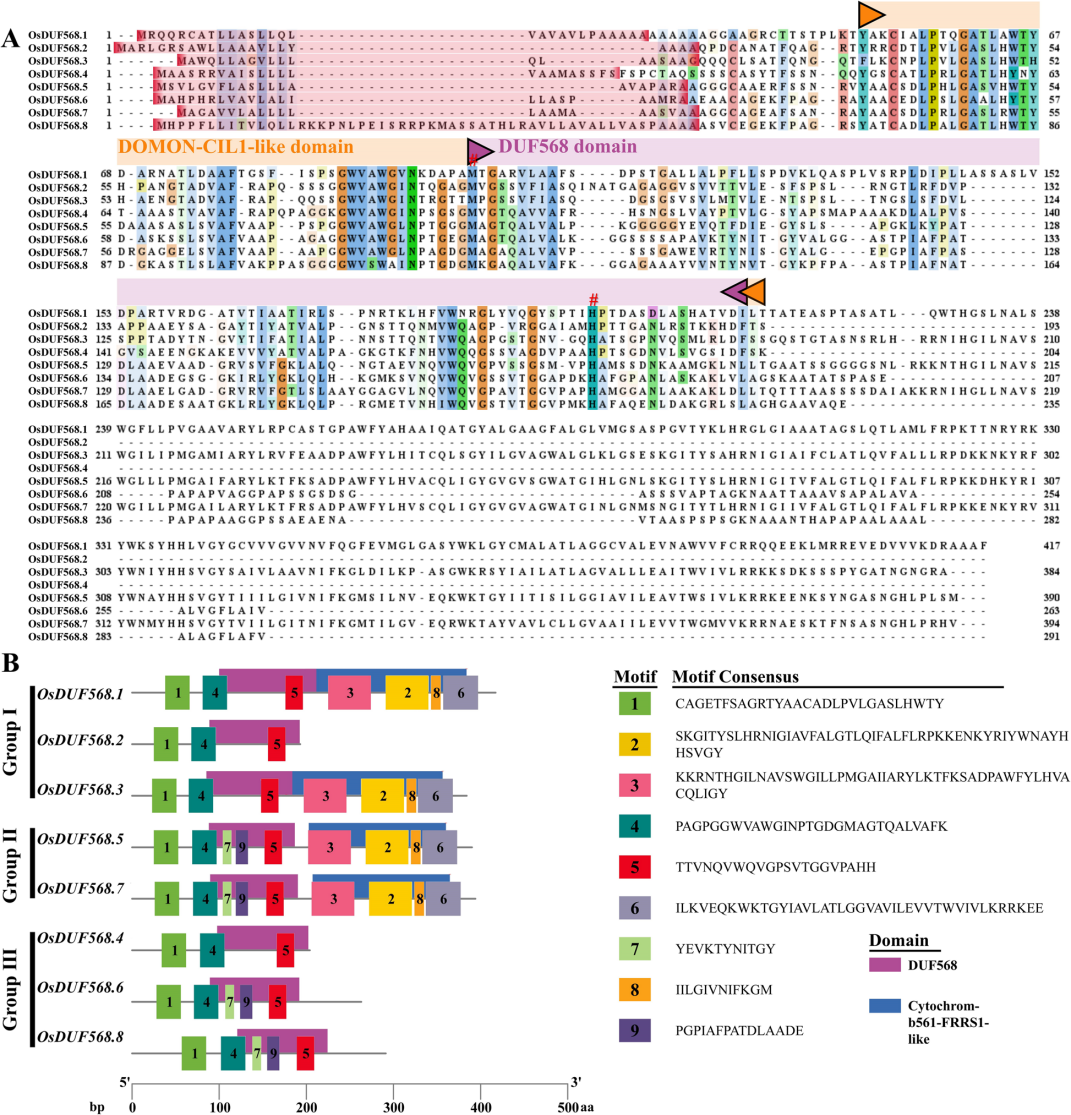
The protein sequences, conserved domains and motifs of OsDUF568. **A** Multiple sequence alignment of OsDUF568 proteins, showing signal peptides, conserved residues, DUF568 domains, and DOMON-CIL1-like domains. Conservative amino acid residues M100 and H197 were labeled with pound signs (#). Signal peptide sequences were shaded in pink. The purple and orange bars represent the DUF568 domain and the DOMON-CIL1-like domain, respectively. **B** Conserved domains and motifs of OsDUF568 proteins. Each domain and motif are illustrated with a specific color, and their distribution corresponds to their positions. The length of genes and proteins can be estimated using the scale at the bottom

Multiple sequence alignment of OsDUF568 proteins was performed using MEGA-X to further understand their homology (Fig. 2A). The conserved amino acid residues (C53, L56, P57, G60, A61, A77, F78, G87, W88, V89, W91, N94, M100, G102, A108, L172, W183, G186, G191, H197) were identified in the range of 40–210, where the DUF568 domain (Purple bar) and DOMON (Dopamine *ß*-monooxygenase redox domains)-CIL1-like domain (Orange bar) were concentrated. M100 and H197 (Fig. 2A pound signs) play a critical role in coordinating the binding of AIR12 (OsDUF568.6 and 8) and heme [11]. All OsDUF568 proteins contained 20–54 amino-acid signal peptides according to the SignalP website (Fig. 2A), and OsDUF568.1, 3, 5, 6 and 7 possessed TM regions base on the TMHMM website (Table S2). Therefore, it is suggested that OsDUF568.1, 3, 5, 6 and 7 proteins may be secreted proteins, while OsDUF568.2, 4 and 8 are not.

To investigate the evolutionary relationships of the *OsDUF568* family, the conserved domains and motifs were analyzed (Fig. 2B). OsDUF568 proteins all have DUF568 domain, OsDUF568.1, 3, 5 and 7 contained Cytochrom-b561-FRRS1-like domain. Further, nine distinct conserved motifs were identified, motifs 1, 4 and 5 were observed in all OsDUF568s, and motifs 4 and 5 surrounded the region of DUF568 domain in all OsDUF568 proteins, which suggested that motifs 4 and 5 may be essential part of the DUF568 domain. Similarly, motifs 2, 3, 6 and 8 may be related to Cytochrom-b561-FRRS1-like domain. Moreover, motifs 7 and 9 were found in OsDUF568s (OsDUF568.4 excluded) belonged to Group II and III. The structural differences of OsDUF568 proteins suggested that OsDUF568 family may have variant specific functions.

### Cis-acting elements of *OsDUF568* genes

**Fig. 3:**
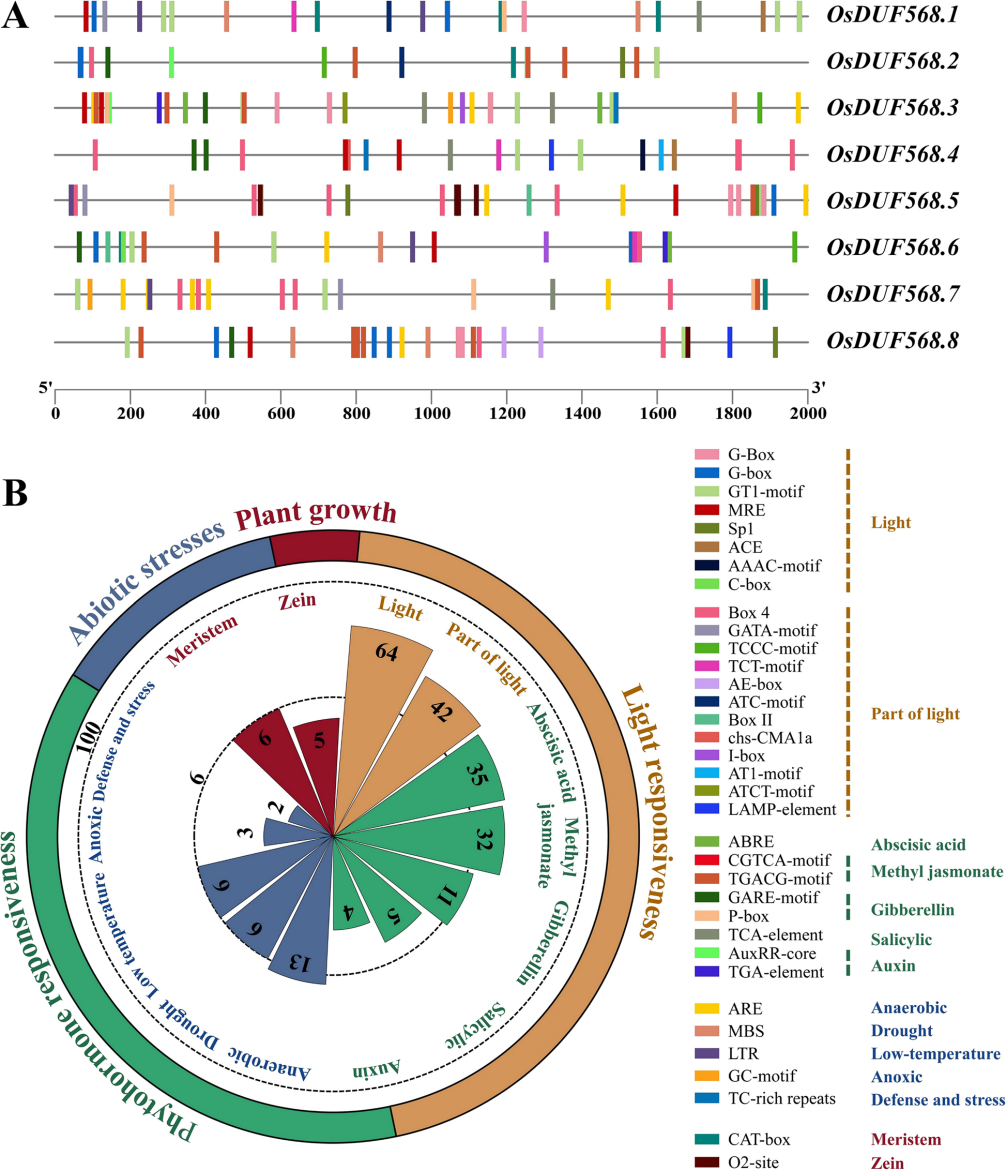
Identification of cis-acting elements in OsDUF568 genes. **A** Distribution of cis-acting elements in 2 Kb upstream of each OsDUF568 gene. The different colored boxes indicate distinct elements. **B** Assessment of different categories and subclasses contained in the OsDUF568 genes. The rose chart on the left shows the proportion of the categories (circle) and subclasses (petal), with the length of each petal proportional to the number of elements. The orange, green, blue and red petals represent light responsiveness, phytohormone responsiveness (including abscisic acid, methyl jasmonate, gibberellin, salicylic acid and auxin), abiotic stress responses (including anaerobic, drought, low-temperature, anoxic, and defense and stress), and plant growth, respectively. The name of the cis-acting elements and their corresponding box colors are sorted from high to low according to their occurrence frequency, accompanied by the subclasses of the cis-acting elements on the right

Analysis of cis-acting elements to a greater detail will facilitate in better understanding the precise control of genes and generate valuable clues for their functional multiplicity [14]. This report manifested potential cis-acting elements in the 2 Kb upstream regions of the *OsDUF568* genes from plantCARE website. Thirty-five cis-acting elements were detected totally (Fig. 3A), which formed four main categories as light responsiveness, phytohormone responsiveness, abiotic stresses and plant growth (Fig. 3B).

The four categories contained eleven subdivisions, the largest subdivision was light responsiveness, which contained 45.3% predicted cis-elements, including G-box (Light-responsive element) and Box 4 (Part of a module for light response) as representatives. A series of regulatory elements participating in plant hormone responsiveness ranked second. Cis-acting factors respond to abscisic acid, methyl jasmonate, gibberellin, salicylic acid and auxin were involved. Among them, ABRE (Related to the abscisic acid response) was covered the largest portion, followed by the TGACG-motif and CGTCA-motif (Methyl jasmonate responsive element). In the abiotic stress response category, ARE (Anaerobic induction element) were the most common, followed by those relating to low-temperature responsiveness (LTR) and drought-inducibility (MBS). As for the plant growth regulation category, only two main stress-related cis-acting factors were identified, known as the CAT-box (Referred to meristem expression) and O2-site (Involved in zein metabolism regulation). Intriguingly, all kinds of cis-regulatory elements distributed widely throughout the promoter regions of *OsDUF568* genes, revealing that *OsDUF568* may have intricate expression patterns and be crucial in the regulation of rice development and stress resistance.

### Expression patterns of *OsDUF568* genes in different tissues and response to plant hormone & abiotic stresses

**Fig. 4:**
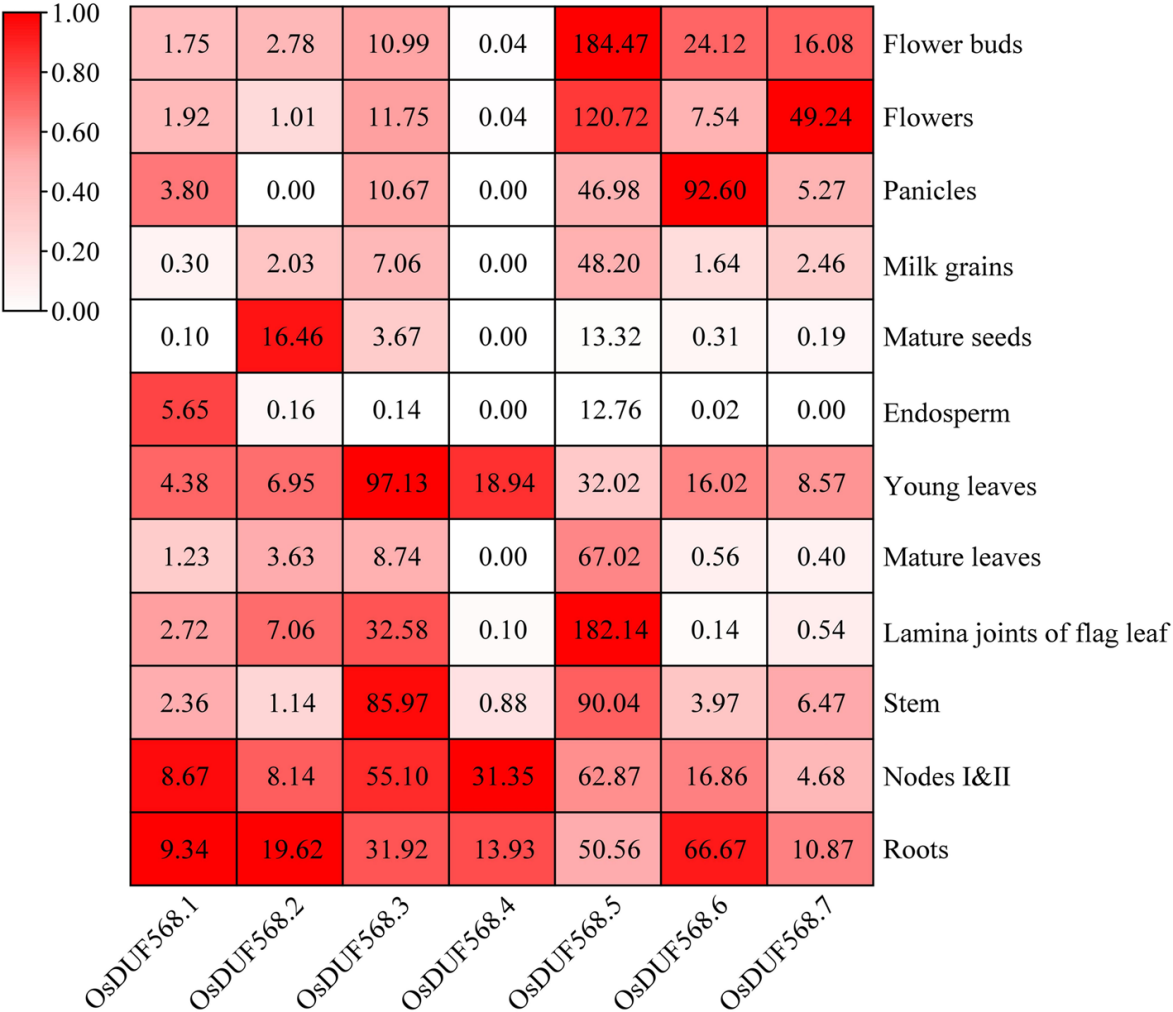
Expression patterns of DUF568 genes in different tissues of rice. Log2 transformed min–max normalized gene expression values were used to generate the heat map. The gene expression levels were quantified as fragments per kilobase per million (FPKM) and visualized as a color gradient in the heat map. The color scale bar on the left side of the heat map represents the relative expression level, ranging from 0 to 1.00, where higher values correspond to the higher expression levels

To further characterize the potential biological function of *OsDUF568* genes (excluding *OsDUF568.8*), the expression patterns were analyzed in 12 different tissues obtained from RiceENCODE website (Fig. 4). The results showed that the expression levels of *OsDUF568* genes varied across different tissues. Specifically, *OsDUF568.3* and *5* were highly expressed in most tissues, while *OsDUF568.1* was expressed in low levels in most tissues. *OsDUF568.4* was barely expressed in nine tissues except for young leaves, nodes I & II, and roots. Importantly, all eight OsDUF568 genes showed high expression levels in these three tissues, indicating that *OsDUF568* genes might be involved in the development of leaves, nodes and roots in rice.

**Fig. 5:**
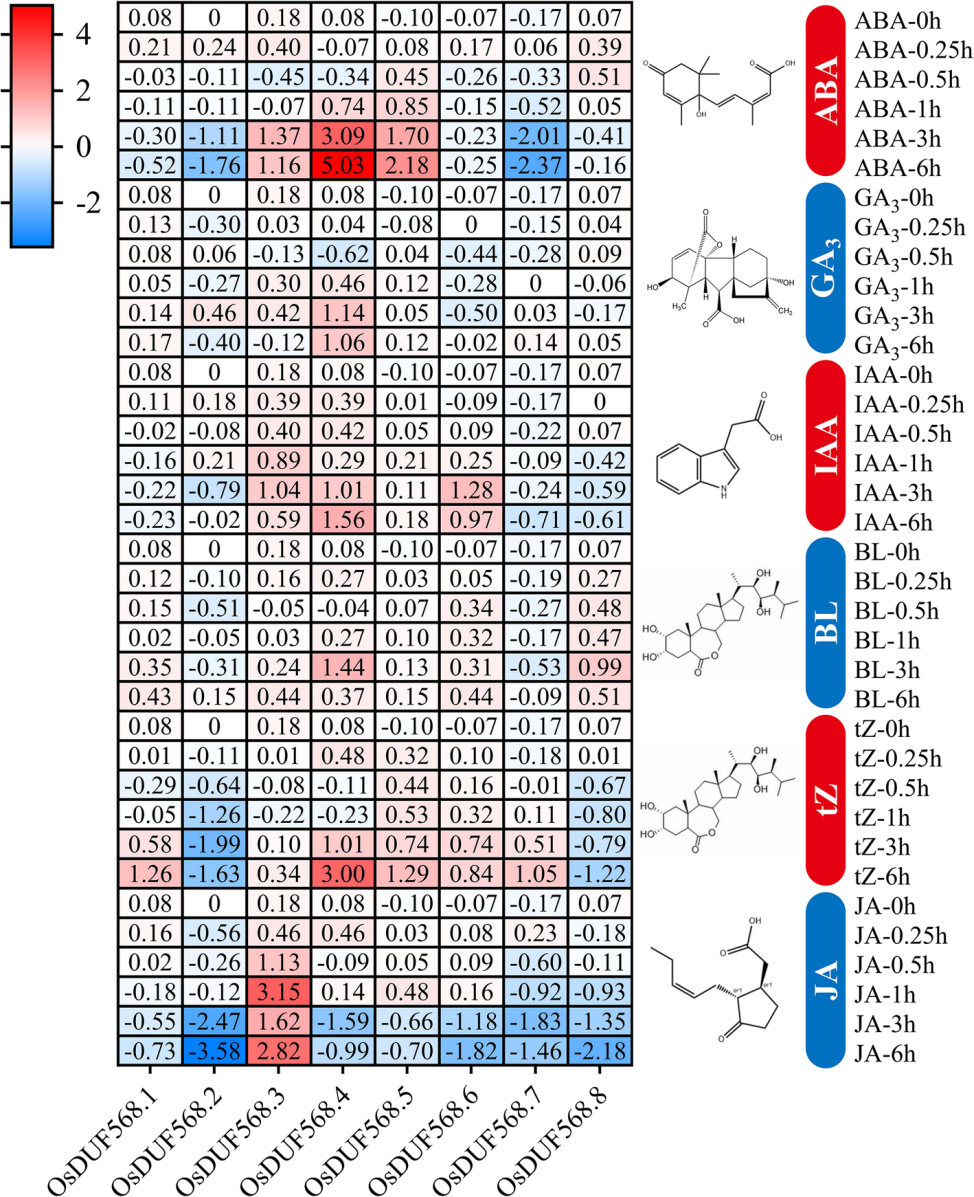
Relative expression of OsDUF568 genes in response to abscisic acid (ABA), gibberellin A_3_ (GA_3_), 3-indoleacetic acid (IAA), brassinolide (BL), trans-zeatin (tZ) and jasmonic acid (JA). The gene expression values were normalized and quantified as Cy5:Cy3 ratios, and visualized as a color gradient in the heat map. The color scale bar on the left represents the relative expression levels, where red indicates up-regulation and blue indicates down-regulation

Notably, there were several plant hormone response elements in the upstream cis-acting elements of *OsDUF568* genes. To further investigate the possible mechanisms of *OsDUF568* genes, this study analyzed the relative expression of *OsDUF568* genes in response to six plant hormones (ABA, abscisic acid; GA_3_, gibberellin A_3_; IAA, 3-indoleacetic acid; BL, brassinolide; tZ, trans-zeatin; JA, jasmonic acid) using the data from the RiceXPro website (Fig. 5). The relative expression of *OsDUF568.4* and *5* were up-regulated after ABA treatment for 3 and 6 h, while *OsDUF568.2* and *OsDUF568.7* were down-regulated. Most *DUF568* genes were insensitive to GA_3_, IAA and BL treatment, with only *OsDUF568.4* showing slight up-regulation after GA_3_, IAA and BL treatment, and *OsDUF568.6* was slightly up-regulated after 3 h of IAA treatment. In addition, *OsDUF568.2* and *8* were down-regulated after tZ treatment, while *OsDUF568.1*, *3*, *4*, *5*, *6* and *7* were up-regulated. Most *OsDUF568* genes were down-regulated after JA treatment, except for *OsDUF568.3*, which showed significant up-regulation. Those results indicated that *OsDUF568* genes might regulate relevant hormone signaling pathways. Notably, the expression of *OsDUF568.4* changed significantly under the treatment of all six plant hormones, suggesting that OsDUF568.4 may be widely involved in rice hormone signaling pathway.

**Fig. 6:**
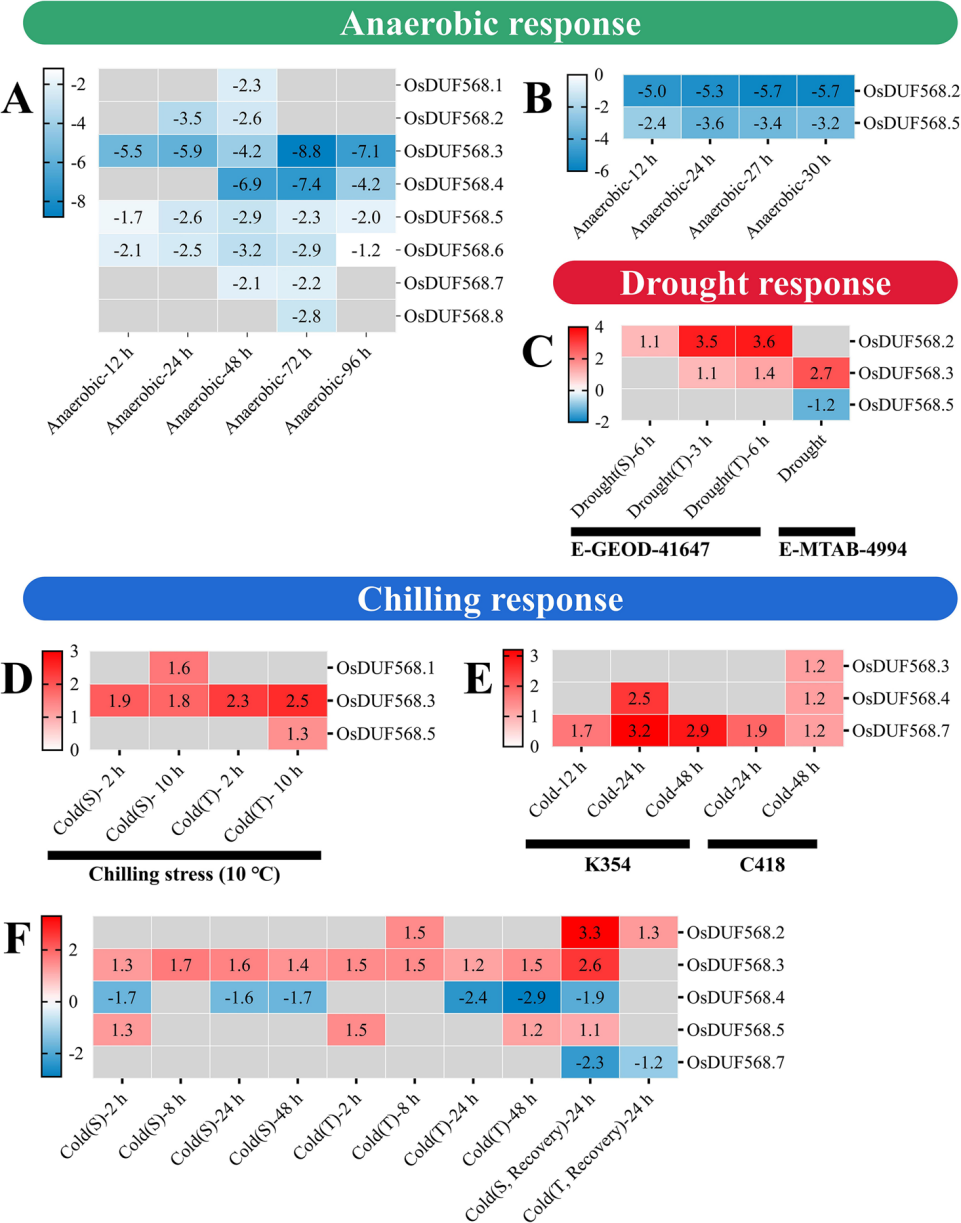
Differential expression of OsDUF568 family genes in response to anaerobic, drought and chilling. **A-B** Fold change in the expression of OsDUF568 genes in rice seeds grown under anaerobic conditions (E-GEOD-115371 and E-MEXP-2267). **C** Differential expression of OsDUF568 genes in susceptible and drought-tolerant rice genotypes under drought conditions (E-GEOD-41647 and E-MTAB-4994). **D-F** Differential expression of OsDUF568 genes in chilling-sensitive and chilling-tolerant genotypes under chilling stress (E-MTAB-5941, E-GEOD-37940 and E-GEOD-38023). Log2-fold change values were used and visualized as a color gradient in the heat maps

There were several cis-acting elements related to abiotic stress response in the upstream regions of *OsDUF568* genes. The changes in gene expression of *OsDUF568* genes in response to various abiotic stresses were analyzed by using publicly available transcriptomic datasets from the Expression Atlas website (Fig. 6).

E-GEOD-115371 [15] and E-MEXP-2267 [16] datasets were analyzed (Fig. 6A&B). The E-GEOD-115371 dataset provided molecular profiling of rice seeds grown under anaerobic conditions for 1 h, 3 h, 12 h, 24 h, 2 days, 3 days, and 4 days [15]. As shown in Fig. 6, all *OsDUF568* genes showed decreased expression in response to anaerobic stress. Among them, *OsDUF568.3* and *4* were strongly down-regulated, reaching the lowest levels at 72 h. The E-MEXP-2267 dataset contained the transcription profiling time course of rice germination under anaerobic conditions, an aerobic to anaerobic switch, and an anaerobic to aerobic switch [16]. *OsDUF568.2* and *5* showed decreased expression in response to anaerobic stress, while no *OsDUF568* gene showed differential expression in response to the switch between aerobic and anaerobic. Overall, our results showed that the *OsDUF568* family were likely to play a role in response to anaerobic stress.

Drought-induced differential gene expression of *OsDUF568.2*, *3* and *5* from two datasets (E-GEOD-41647 and E-MTAB-4994) were showed (Fig. 6C). E-GEOD-41647 contained data for *OsDUF568.2* and* 3* from seedlings of susceptible IR20 and drought-tolerant Dagad deshi genotypes [17]. Both *OsDUF568.2* and* 3* were up-regulated in Dagad deshi, but only *OsDUF568.2* in IR24. Moreover, *OsDUF568.2* was more up-regulated in Dagad deshi than in IR24, and its expression level increased with drought duration in Dagad deshi. The E-MTAB-4994 dataset contained data for *OsDUF568.3* and *5* from the flag leaf at the panicle initiation stage of Nagina 22 (a drought-tolerant genotype) [17]. *OsDUF568.3* showed up-regulation while *OsDUF568.5* showed down-regulation in response to drought in Nagina 22. The results indicated that *OsDUF568* family played a role in response to drought stress.

To understand the role of *OsDUF568* family members in chilling/cold tolerance and susceptibility, three datasets (E-MTAB-5941, E-GEOD-37940 and E-GEOD-38023) were analyzed (Fig. 6D, E, F). E-MTAB-5941 contained the data on short- and long-term stress-induced changes in the transcriptome of a chilling-sensitive genotype Thaibonnet and a chilling-tolerant genotype Volano, each subjected to 2 and 10 h chilling treatment at 10 °C [18]. *OsDUF568.3* was up-regulated in Thaibonnet and Volano, while *OsDUF568.1* and *5* were only up-regulated in Thaibonnet and Volano, respectively. E-GEOD-37940 comprised the transcriptome of the cold tolerant introgression line K354 and its recurrent parent C418 under cold stress [19]. OsDUF568.4 and 7 were up-regulated in K354 and C418, while OsDUF568.3 was up-regulated in C418 only. E-GEOD-38023 contained expression data from a chilling-tolerant Li-Jiang-Xin-Tuan-He-Gu (LTH) *japonica* landrace variety and a chilling-sensitive IR29 *indica* cultivar. The plants from both genotypes were subjected to chilling treatment at 4 ℃, and then moved to normal temperature 29 ℃ for 24 h to allow recovery [20]. *OsDUF568.2*, *3* and *5* were up-regulated in LTH and IR29, while *OsDUF568.4* was down-regulated. *OsDUF568.7* showed down-regulation in response to normal temperature for recovery. In general, *OsDUF568* family genes were differentially regulated in response to chilling between the tolerant and the susceptible rice genotypes.

### Co-expression gene networks of *OsDUF568* genes

**Fig. 7:**
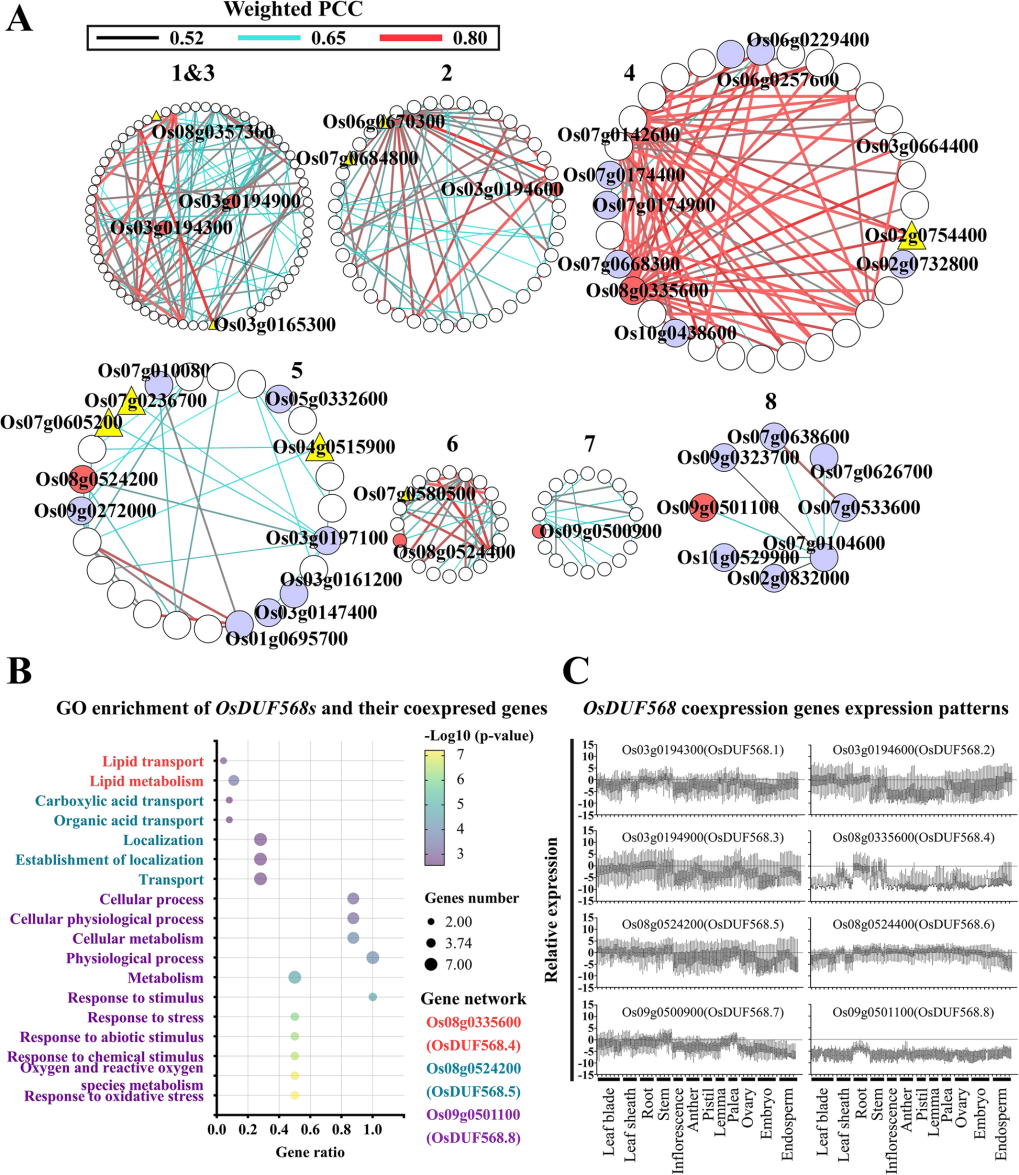
Co-expressed gene networks analysis of OsDUF568 genes. **A** Co-expressed gene networks of DUF568 genes. The red circles represent OsDUF568 genes, the purple circles represent genes from gene ontology (GO) enrichment, and the yellow triangles represent transcription factors. Weighted Pearson correlation coefficients (PCC) are represented by lines, with values close to 0.52 shown as thin black lines, values close to 0.65 shown as blue lines, values close to 0.80 shown as thick red lines. **B** GO enrichment analysis (Biological process) of OsDUF568s and their co-expressed genes. GO enrichment was found in OsDUF568.4, 5 and 8 networks. **C** OsDUF568 co-expression genes expression patterns. Data came from RiceXPro, which was performed 75 percentile normalization with log2 transformation and the relative expression value (log2) was obtained by subtracting the median expression value within the data set for each probe

Co-expression network analysis of *OsDUF568* genes has the potential to reveal the putative functions of the genes involved in biological processes [21]. The co-expression gene networks of *OsDUF568* genes were constructed using the RiceFREND website (Fig. 7A and Table S3). The Weighted Pearson correlation coefficient (PCC) of genes in most networks was around 0.65, while the genes in the network of *Os08g0335600* (*OsDUF568.4*) network had a higher coefficient, suggesting a close functional relationship between the genes in this network. In addition, some genes were co-expressed with both *Os03g0194300* (*OsDUF568.1*) and *Os03g0194900* (*OsDUF568.3*), and most of these genes were related to enzymes such as endoglucanase, caffeic acid 3-O-methyltransferase, and transferase.

Gene ontology (GO) enrichment analysis was performed to analyze the co-expressed networks of *OsDUF568* genes (Fig. 7B). Effective results were obtained for *OsDUF568.4, 5* and* 8*. Network *Os09g0501100* (*OsDUF568.8*) showed significant enrichment with GO terms related to metabolic and stress response, such as ‘oxygen and reactive oxygen species metabolism (GO:0006800)’, ‘response to chemical stimulus (GO:0042221)’, ‘response to oxidative stress (GO:0006979)’ and ‘response to abiotic stimulus (GO:0009628)’ (Fig. 7B). The relative expression of network *Os09g0501100* (*OsDUF568.8*) was high only in roots (Fig. 7C), suggesting that *OsDUF568.8* and its co-expressed genes may affect the metabolic and stress response of rice roots. Network *Os08g0524200* (*OsDUF568.5*) mainly consisted of ‘Carboxylic acid transport (GO:0046942)’, ‘Organic acid transport (GO:0015849)’ and ‘Localization (GO:0051179)’, while network *Os08g0335600* (*OsDUF568.4*) contained ‘Lipid transport (GO:0006869)’ and ‘Lipid metabolism (GO:0006629)’ (Fig. 7B). The networks of *OsDUF568.4* and* 5* were related to transport and highly expressed in leaves, stems and roots (Fig. 7C), indicating that they may participate in the transport of substances during the vegetative growth period of rice.

Remarkably, the co-expressed genes of *OsDUF568* networks were highly expressed in roots, stems and leaves (Fig. 7C), which was consistent with the expression patterns of *OsDUF568* genes (Fig. 4), suggesting that *OsDUF568* and co-expressed genes may play important roles in rice development.

### Protein–protein interaction networks analysis of OsDUF568 proteins

**Fig. 8:**
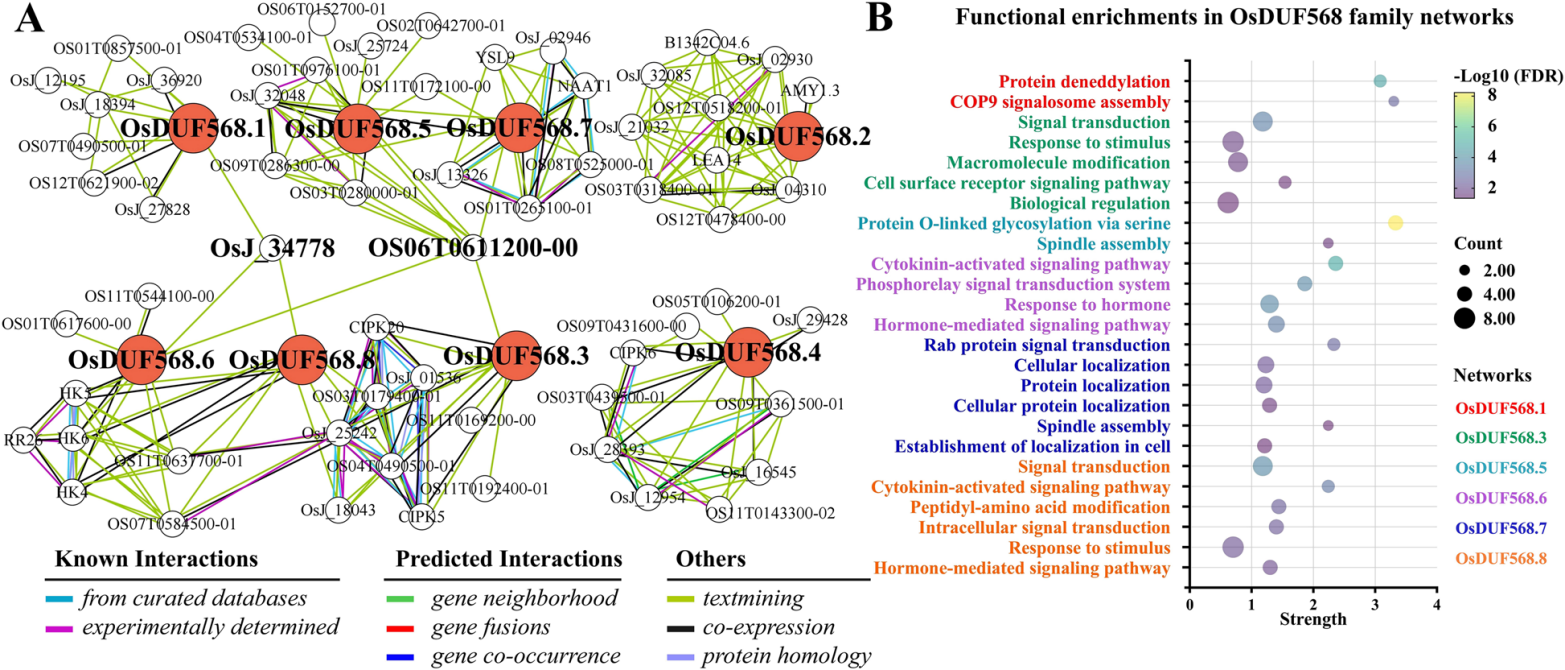
Protein–protein interaction analysis of OsDUF568 proteins. **A** Interaction network of OsDUF568 proteins. Red circles represent OsDUF568 proteins and white circles represent predicted functional partners of OsDUF568s. Different colored edges represent different protein–protein associations. **B** Functional enrichment analysis (Biological process) of OsDUF568s and their interacting proteins with FDR < 0.05

The predicted functional partners of OsDUF568s were identified from STRING website, and the protein–protein interaction (PPI) networks were constructed (Fig. 8A and Table S4). The association of most proteins in the OsDUF568 PPI networks were textmined. In addition, there were a number of co-expressed and experimentally determined associated proteins in the networks of OsDUF568.6 and 8. Some proteins were associated with multiple OsDUF568 proteins. Among them, Auxin-repressed protein-like protein ARP1 (OsJ_34778) was associated with OsDUF568.1, 6, and 8, and Pentatricopeptide (PPR) repeat-containing protein-like protein (OS06T0611200-00) was associated with OsDUF568.3, 5, 6 and 7, which indicated the function of the OsDUF568 family may be closely related to these two proteins.

According to functional enrichments (Fig. 8B), most networks of OsDUF568 family were related to signal transduction, and proteins in OsDUF568.6 and 8 networks were related to hormone-mediated signaling pathway, especially cytokinin-activated signaling pathway (GO:0009736). Meanwhile, proteins in OsDUF568.1 networks were related to protein deneddylation (GO:0000338) and COP9 signalosome (CSN) assembly (GO:0010387). CSN complex regulates the activity of cullin-RING ligase (CRL) families of E3 ubiquitin ligase complexes, and play critical roles in regulating gene expression, cell proliferation, and cell cycle [22]. The functions of proteins in OsDUF568.7 may include Rab protein signal transduction (GO:0032482) and Cellular localization (GO:0051641), Rab proteins affect cell growth, motility and other biological processes [23]. The results indicated that OsDUF568 family may be involved in material transportation, metabolism and signal transduction in rice.

### Expression of OsDUF568 family in response to phytohormones and abiotic stresses

**Fig. 9:**
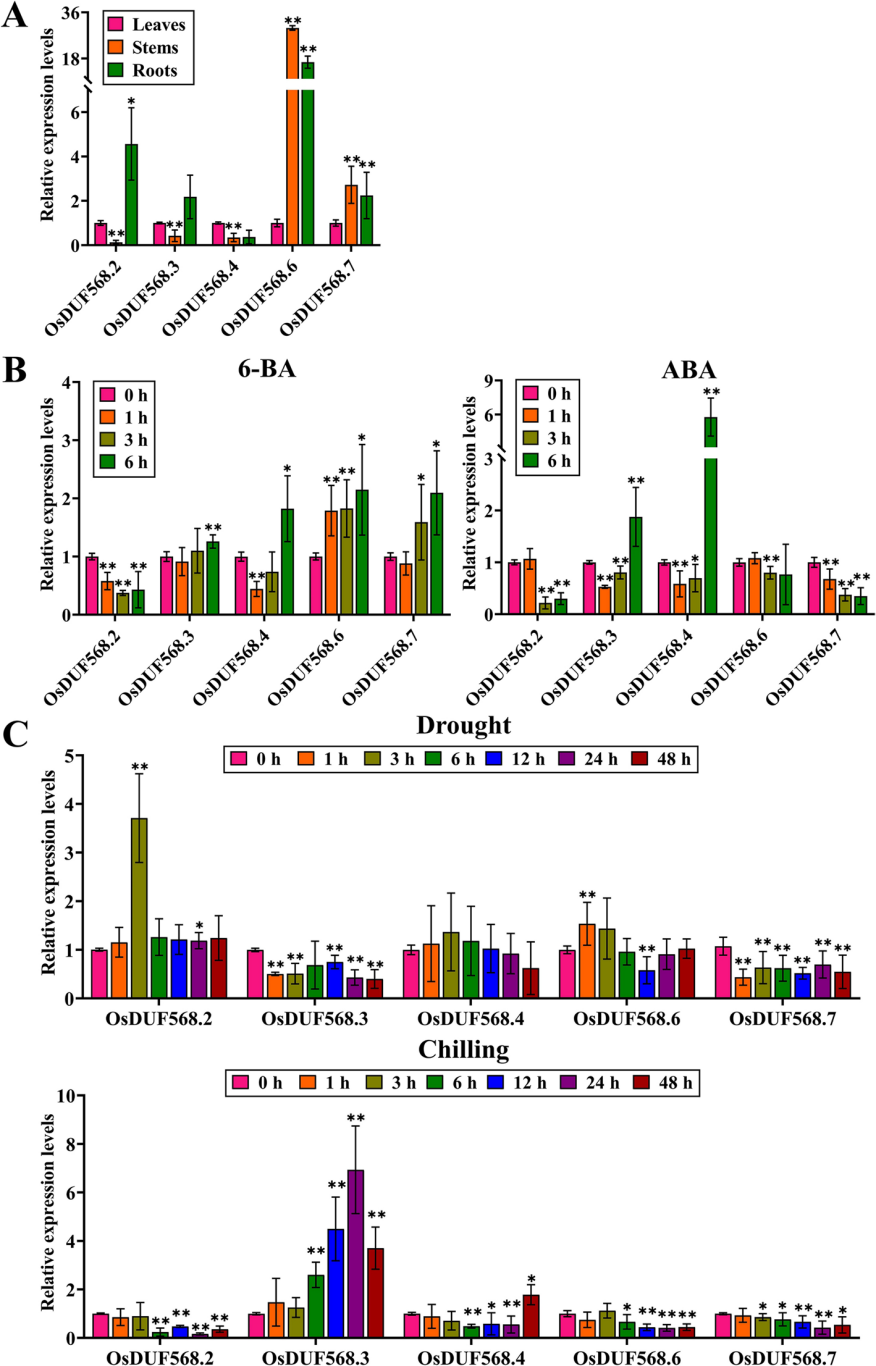
Expression profile analysis of OsDUF568 genes in rice seedlings. Relative expression level of OsDUF568 genes in various tissues at rice seedlings (**A**). Relative expression level of OsDUF568 genes under 6-BA and ABA treatments (**B**). Relative expression level of OsDUF568 genes under drought and cold treatments (**C**). Error bars represent ± SD. * and ** indicate the significant difference according to Student’s t-test

The published data from several public databases above showed that some *OsDUF568* family members had higher expression levels in different rice tissues, and were repressed under multiple phytohormones and abiotic stresses, to confirm experimentally, the *OsDUF568.2*,* 3*,* 4*, *6* and* 7* expression in rice seedlings subjected to various phytohormones (ABA and 6-BA) and abiotic stresses (drought and cold) treatments were examined. The expression of *OsDUF568* genes in leaves, stems and roots of rice seedlings were investigated (Fig. 9A). The *OsDUF568.2*, *3* and *4* transcript level in stems were lower than leaves and roots, while *OsDUF568.6* and *7* were higher. Furthermore, *OsDUF568.2*, *3*, *6* and *7* is highly expressed in the roots. The results suggested the expression of *OsDUF568* genes exhibited significant tissue specificity in rice.

The expression of *OsDUF568* genes were also regulated by phytohormones. As shown in Fig. 9B, the expression level of *OsDUF568.2* decreased after 6-BA treatment, while *OsDUF568.3*, *4*, *6* and *7* reached their highest level after 6-BA treatment for 6 h. Under ABA treatment (Fig. 9B), the expression level of *OsDUF568.2* and *7* were suppressed gradually, *OsDUF568.3* and 4 were induced to reach highest level after 6 h treatment.

*OsDUF568* genes showed significant response to abiotic stresses. *OsDUF568.2* expression level reached highest at 3 h after drought treatment, then descend. Expression of *OsDUF568.3*, and *7* were gradually repressed by drought treatments (Fig. 9C). *OsDUF568.6* expression level was induced slightly at the initial time point and suppressed to the lowest level at 12 h. As to cold stress (Fig. 9C), the *OsDUF568.2*, *4*, *6* and *7* expression level were rapidly suppressed. On the contrary, *OsDUF568.3* expression level for cold stress was first induced and then suppressed again. These results indicated that *OsDUF568* genes were involved in responses to multiple phytohormones and abiotic stresses.

## Discussion

According to the RAP-DB, NCBI (National Center for Biotechnology Information) websites, and results from Preger et al. [11] and us, OsDUF568 family was found to contain DOMON-CIL1-like and Cytochrom-b561-FRRS1-like domains. The DOMON superfamily may be a direct participant in the electron transfer process [24]. Cytochromes b561 (CYB561s) are a family of di-heme transmembrane (TM) proteins that use ascorbate (ASC) as an electron donor and are present in various organs and cell types in plants and animals. The CYB561-core domain is associated with DOMON in ubiquitous CYBDOM proteins, which comprise a novel electron-transfer system potentially involved in oxidative modification of cell-surface proteins. CYB561s and CYBDOMs play important roles in plants such as stress defense, cell wall modifications and cell metabolism [25]. In addition, AIR12s (OsDUF568.6 and 8) were also found to be involved in the establishment of a redox connection between the cytoplasm and the apoplast [11]. Further, the OsDUF568 proteins also contained multiple conserved amino acids (Fig. 2A), with methionine and histidine supporting the binding of OsDUF568 proteins and hemes [11]. All results above showed OsDUF568 family may be involved in stress defense and cell metabolism by mediating electron transport of redox domains in rice.

The upstream regions of *OsDUF568* genes were found to contain cis-acting elements that respond to light, phytohormone, and abiotic stresses (Fig. 3), suggesting that these factors may interact to regulate the expression of *OsDUF568* genes. The expression patterns of *OsDUF568* were investigated in 12 tissues (Fig. 4), and it was found that these genes were highly expressed in rice roots, stems and nodes I and II, particularly in roots, suggesting their importance in rice growth and root development. Furthermore, the relative expression of *OsDUF568* genes under six hormone treatments showed that *OsDUF568* genes were sensitive to ABA, tZ and JA treatments (Fig. 5). ABA treatment significantly altered the expression of four *OsDUF568* genes, while tZ treatment up*-*regulated most of *OsDUF568* genes, and JA treatment down-regulated most. The results indicated that *OsDUF568* genes may participate in these hormone pathways in rice.

Rice is adversely affected by abiotic stresses including anaerobic [26, 27], drought [28] and cold [19, 20]. Several reports showed DUFs may be important for rice resistance to abiotic stresses [7–9]. All *OsDUF568* genes showed decreased expression in response to anaerobic stress (Fig. 6A), indicating that *OsDUF568* family were likely to play a role in response to anaerobic stress. Besides, *OsDUF568.2* and *OsDUF568.3* positively responded to drought stress (Fig. 6B), elucidating their roles in rice adaption to the drought environment. Similarly, *OsDUF568.1*, *2*, *3*, and *5* were up-regulated in chilling stress (Fig. 6C), suggesting the four *OsDUF568* genes may be important for rice resistance to cold. Considering the common positive response of *OsDUF568.2* and *OsDUF568.3* under drought and chilling stresses, overexpression of the two genes in rice may be effective methods to engineering plant fitness for drought and cold conditions.

The co-expression (Fig. 7) and PPI (Fig. 8) networks of *OsDUF568* genes were constructed, and the possible function of these genes were studied using GO enrichment analysis. The results showed that *OsDUF568* genes were widely involved in material transportation, metabolism and signal transduction in rice. Meanwhile, AIR12 (OsDUF568.6 and 8) may be related to hormone-mediated signaling pathway, like cytokinin. *OsDUF568.6* was up-regulated while *OsDUF568.8* was down-regulated after trans-zeatin treatment, and the two genes showed different expression in response to other phytohormone (Fig. 5). The results indicated the function of AIR12 protein was closely related to phytohormone signal transduction, especially cytokinin-activated signaling pathway.

To understand the potential biological functions of *OsDUF568* genes in rice, the RNA transcript levels of *OsDUF568.2*,* 3*,* 4*, *6* and* 7* genes in different rice tissues, and treated by phytohormones and abiotic stresses were further investigated in rice seeding (Fig. 9). *OsDUF568* genes were generally highly expressed in rice roots, which was consistent with the predicted results (Fig. 4). This indicated that *OsDUF568* family may be vital for the development of rice roots. Expression analysis revealed that some *OsDUF568* genes were induced or inhibited by different phytohormones treatment. Among them, *OsDUF568.4* and* 6* were significantly induced after 6-BA treatment. Previous reports showed that *OsDUF568.6* would be induced after cytokinin treatment. In addition, cytokinin-inducible type-A response regulator OsRR6 acted as a negative regulator of cytokinin signaling, *OsDUF568.4* was highly expressed in rice transgenic lines overexpressing *OsRR6* [29]. This suggests that *OsDUF568.4* and *6* may be involved in cytokinin signaling pathway. Besides, *OsDUF568.3* and *4* were upregulated after both 6-BA and ABA treatment, which suggested *OsDUF568.3* and *4* may participate in both abscisic acid and cytokinin signaling pathways. The expression levels of most *OsDUF568* genes were decreased under drought and cold stress treatments, while *OsDUF568.2* and *3* were significantly induced under drought and cold treatments, respectively. *OsDUF568.2* has previously been reported as a gene within a quantitative trait locus (QTL) region for high grain yield under lowland drought [30]. Further research on the biological functions of OsDUF568.2 may be helpful to develop drought resistant versions of popular varieties.

Taken together, these results indicated that *OsDUF568* gene family was essential for the development of leaves, stems and roots of rice. The OsDUF568 family may also participate in abscisic acid and cytokinin signaling pathways, and be related to abiotic stress resistance in those vegetative tissues of rice.

## Conclusions

This study conducted a comprehensive analysis of the OsDUF568 family. The phylogenetic tree showed a close evolutionary relationship between *DUF568* members in rice and maize, while those in *Arabidopsis* were distantly related. Cis-element prediction displayed that over 82% of the elements upstream of *OsDUF568* were responsive to light and phytohormones. Expression patterns revealed that all 7 *OsDUF568* genes searched were highly expressed in young leaves, nodes I and II, and roots of rice. Furthermore, the expressions of some *OsDUF568* genes were responsive to plant hormones (abscisic acid, trans-Zeatin and jasmonic acid) and abiotic stress (anaerobic, drought and chilling). Further GO analysis of the co-expression and PPI networks revealed that *OsDUF568* related genes were enriched in material transportation, metabolism and signal transduction in rice. Finally, RT-qPCR experiments indicated that *OsDUF568* family was highly expressed in rice roots, and may participate in signaling pathways involved in phytohormones and abiotic stresses. The findings provide valuable insights into the OsDUF568 family and contribute to the elucidation of their biological functions in the future.

## Methods

### Identification of *DUF568* gene family members and phylogenetic analysis

The HMM (Hidden markov model) of the DUF568 (PF04526) domain was obtained from Pfam [31]. The HMM was compared with the whole protein sequences of rice, *Arabidopsis thaliana* and maize obtained from the NCBI [32] using HMMER ver. 3.0 (E-value < 10^–15^) [33]. The MSU and RAP loci of *DUF568* genes in rice were obtained from the China Rice Data Center [34]. Exons and chromosome locations were obtained from the NCBI [32], and description were obtained from RAP-DB (The Rice Annotation Project Database) [35]. Protein physicochemical properties were analyzed using the ProtParam [36], and subcellular localization was predicted using the PSORT [37].

The full length DUF568 protein sequences of rice, *Arabidopsis* and maize were compared in MEGA-X. Multiple sequence alignment was performed using the MUSCLE aligner with all other parameters set to the default settings. The neighbor joining tree was constructed using the bootstrap method with 1000 repetitions.

### Comparison of protein sequence, gene structure, conserved domains and motifs

The amino acid sequences of OsDUF568 proteins in rice were compared using the MUSCLE method in MEGA-X and visualized using Jalview. The signal peptides were identified using the SignalP [38], and the transmembrane regions were analyzed using the TMHMM [39]. The conserved motifs of OsDUF568 proteins were predicted using the MEME [40], domains were obtained from Pfam, and both were visualized using TBtools [41].

### Cis-acting elements

The genome annotation for rice was obtained from NCBI [42]. The cis-acting elements located within 2 Kb upstream of the *OsDUF568* genes were extracted using the plantCARE website [43] and visualized using TBtools [41]. The rose chart was created using Microsoft Office PowerPoint 2019.

### Expression patterns

The expression patterns of *OsDUF568* genes in 12 different tissues (including flower buds, flowers, panicles, milk grains, mature seeds, endosperm, young leaves, mature leaves, lamina joints of flag leaf, stem, nodes I and II, and roots) were obtained from the RiceENCODE [44]. The expression patterns in response to plant hormones (including ABA, GA_3_, IAA, BL, tZ and JA) were obtained from the RiceXPro [45]. Gene expression data for *OsDUF568* genes under abiotic stress conditions were extracted from several datasets available at the EMBL-EBI Expression Atlas website [17] including E-GEOD-115371, E-MEXP-2267, E-GEOD-41647, E-MTAB-4994, E-MTAB-5941, E-GEOD-37940 and E-GEOD-38023. Log2-fold change values were used and visualized as a color gradient in the heat maps. All data were visualized using TBtools [41].

### Co-expressed genes and PPI networks

The co-expressed genes of *OsDUF568* genes were searched using RiceFREND [46], Gene ontology (GO) terms were obtained from the GO [47] with P < 0.05 and FDR (False discovery rate) < 0.05. The expression patterns of co-expressed networks in different rice tissues were obtained from the RiceXPro website [48], and visualized using R. The PPI networks and functional enrichments results of OsDUF568 proteins were obtained from STRING [49]. Both networks were drawn by Cytoscape ver. 3.9.0 [50].

### Phytohormone treatments and abiotic stress treatments

The rice cultivars japonica Nipponbare was used for all RT-qPCR analysis. The rice seeds were soaked in 75% alcohol for 1 min, 20% Sodium hypochlorite for 15 min for surface disinfection, and clean the seeds 10 times with water. The seeds were soaked in a fresh water at 28 ℃ for 24 h and germinate for 24 h at 37 °C. Germinated seedlings were transferred to a IRRI (International rice research institute) hydroponic system. Plants were grown in a growth-chamber at 30 °C / 25 °C in a 16-h-light / 8-h-dark cycle and with 75% humidity.

7-day-old seedlings were used to examine the expression patterns of *OsDUF568.2*, *3*, *4*, *6* and *7*. The leaves, stems and roots were sampled from seedlings without any treatment. For drought stress, the roots of seedlings were immersed in 15% PEG-6000 for drought stress. For cold stress, seedlings were transferred to a growth chamber at 4 ℃. The various treated roots were sampled at 0, 1, 3, 6, 12, 24 and 48 h after the abiotic stresses. Phytohormone treatments were performed by adding into the hydroponic system with 50 μM abscisic acid (ABA) and 1 μM 6-benzylaminopurine (6-BA) respectively, and then the roots were sampled at 0, 1, 3 and 6 h after phytohormone treatments. These collected samples were immediately frozen in liquid nitrogen and stored at -80 ℃. Three replications were performed.

### Isolation of RNA, real-time quantitative PCR and expression analysis

Total RNA was isolated from the collected samples using the RNApure Plant kit (CWBIO, Nanjing, China). Using ToloScript All in one RT EasyMix for qPCR (TOLOBIO, Nanjing, China) to remove residual genomic DNA and synthesize first-strand cDNA. RT-qPCR was performed with 2 × Q3 SYBR qPCR Master mix (TOLOBIO, Nanjing, China) in a final reaction volume of 10 μL using an Bio-Rad CFX Connect Real-Time PCR Instrument (Bio-Rad, Bio Rad, Hercules, USA). OsActin (Gene ID: 4333919) served as internal controls. Expression levels are depicted as cycle threshold (Ct) value of the candidate gene relative to the Ct value of the housekeeping gene. Data were analyzed with the Bio-Rad CFX Manager software and visualized using R. All gene-specific primers are listed in Table S5.

### Supplementary Information

**Additional file 1: Table S1.** DUF568 family in Arabidopsis and maize.

**Additional file 2: Table S2.** Trans-membrane regions prediction of OsDUF568 family.

**Additional file 3: Table S3.** Co-expressed genes of the OsDUF568 family and gene-ontology enrichment analysis.

**Additional file 4: Table S4.** PPI networks of the OsDUF568 family and functional enrichment analysis.

**Additional file 5: Table S5.** Gene-specific primers used for RT-qPCR experiments.

## Data Availability

All data generated or analyzed during this study are included in this article and its additional files. All data of the co-expressed genes and potential interacting proteins of the OsDUF568 family obtained based on the open databases, and the results of the GO enrichment analysis were available in Table S3 and S4.

## Abbreviations

ABA: Abscisic acid
AIR12: Auxin induced in root cultures
ARE: Anaerobic induction induction element
ASC: Ascorbate
BL: Brassinolide
CYB561s: Cytochromes b561
DUF: Domains of unknown function
GA_3_: Gibberellin A3
GO: Gene ontology
GRAVY: Grand average of hydropathicity
IAA: 3-Indoleacetic acid
II: Instability index
JA: Jasmonic acid
LTH: Li-Jiang-Xin-Tuan-He-Gu
LTP: Lipid transfer protein
LTR: Low-temperature responsiveness
MBS: Drought-inducibility
NCBI: National center for biotechnology information
pI: Isoelectric points
PPI: Protein-protein interaction
QTL: Quantitative trait locus
RAP-DB: The rice annotation project database
RR: Response regulator
RT-qPCR: Real-time quantitative PCR
TM: Trans-membrane
tZ: Trans-zeatin
